# Evolving student health priorities and school-based health-promoting interventions in Quebec, 2016–2025

**DOI:** 10.24095/hpcdp.46.7/8.03

**Published:** 2026-07

**Authors:** Robert J. Wellman, Erin K. O’Loughlin, Annie Montreuil, Mounia Naja, Jennifer L. O’Loughlin

**Affiliations:** 1 Department of Population and Quantitative Health Sciences, Division of Preventive and Behavioral Medicine, UMass Chan Medical School, Worcester, Massachusetts, USA; 2 Centre de recherche du centre hospitalier de l’Université de Montréal (CRCHUM), Montréal, Quebec, Canada; 3 Faculty of Kinesiology and Physical Education, University of Toronto, Toronto, Ontario, Canada; 4 Institut national de santé publique du Québec, Montréal, Quebec, Canada; 5 Department of Social and Preventive Medicine, Université de Montréal, Montréal, Quebec, Canada

**Keywords:** school health services, health inequalities, primary schools, health promotion

## Abstract

**Introduction:**

The World Health Organization (WHO) recommends implementing health-promoting interventions (HPIs) in schools to enhance student health and well-being. The repeat cross-sectional Prome*SS* project examines whether social inequalities are evident in perceived priority of student health concerns and in HPI availability in Quebec public schools.

**Methods:**

We analyzed data from 171 primary (K–6) schools in Prome*SS* I (2016–2019) and 218 in Prome*SS* II (2023–2025), which updates findings post-COVID-19 and explores additional priorities. Principals or designated staff documented priority student health-related concerns and availability of related HPIs in structured interviews (Prome*SS* I) or online/paper questionnaires (Prome*SS* II). Data analysis emphasized descriptive comparisons.

**Results:**

Perceived importance increased for unhealthy eating (+16%), inadequate sleep (+15%), physical inactivity (+13%), and mental health (+12%). HPI availability increased for mental health (+12%) and personal safety (+13%) but declined for physical activity (–13%) and dental health (–19%), despite government mandates. In 2023–2025, screen time was a universal concern (98%), but only three schools offered related HPIs. Sedentary behaviour was a common concern (51%), with 42% HPI availability. Three-quarters of schools implemented HPIs related to unsafe use of technology. Key challenges to HPI availability included insufficient staff (62%), funding (45%), and implementation support (35%). Few schools reported low engagement from staff, students, or families.

**Conclusion:**

From 2016 to 2025, concerns about student health grew, especially for mental health, inactivity, and nutrition. Yet HPI availability remains inconsistent. Increasing financial resources and implementation support is critical to align school health efforts with evolving needs.

## Highlights

• Schools reported rising concern about unhealthy eating, sleep, physical inactivity, and mental health between 2016 and 2025.

• HPI availability increased for mental health and personal safety but declined for government-mandated physical activity and dental health.

• Screen time and trauma exposure were widely identified as important but often lacked corresponding HPIs.

• Learning difficulties were nearly universally reported, possibly reflecting lasting post-pandemic academic impacts.

• Key barriers to HPI implementation included lack of staff time (62%), funding (45%), and implementation support (35%). Few schools reported low engagement from staff, students, or families, suggesting strong community involvement.

## Introduction

To enhance health and well-being among school-aged children, WHO’s Health-Promoting Schools framework promotes supportive physical and social environments, age-appropriate health education, policies fostering respectful climates, and partnerships among schools, families, communities, and government, emphasizing access to well-being support services.^1^^,^^2^ School-based health-promoting interventions (HPIs) are key within this framework, helping to address health inequalities rooted in socioeconomic disparities^3^ and improve child and adolescent outcomes.^4^ To amplify impact, WHO encourages schools to integrate HPIs with policy, systems, and environmental (PSE) strategies (i.e. structural changes that make healthy choices accessible). PSE examples include providing nutritious meals; investment in physical activity resources, space, and staff; and free in-school health and dental programs.^5^

Quebec supports HPI and PSE uptake through its ÉKIP (Health, Well-Being and Educational Success of Young People) framework.^6^ Examples of PSE implementation in Quebec schools include “Positive Intervention”, promoting positive mental health in young children in the Eastern Townships,^7^ and “How can we do better?”, to prevent problems related to diet, physical inactivity, and obesity.^8^ In 2016, with support from the Ministry of Health and Social Services, we launched Prome*SS,* a repeat cross-sectional study informed by Rogers' Diffusion of Innovations Theory,^9^ to examine social inequalities in perceived priority of student health concerns and HPI availability in Quebec public schools. Prome*SS* is one of the rare studies examining multiple HPIs province wide.

In Prome*SS* I (2016–2019), administrators in disadvantaged primary (K–6) schools were more likely than those in advantaged schools to prioritize lack of physical activity, unhealthy eating, poor hygiene, and dental health. However, only mental-health HPIs were implemented less frequently in disadvantaged schools.^10^ HPIs addressing physical activity, bullying/cyberbullying/exclusion, sexual health, and dental health (all mandated by the Ministry of Education) were equally available across schools. These findings highlight the importance of monitoring HPI presence and equity across socioeconomic contexts.

Prome*SS* II commenced in 2023 to update Prome*SS* I findings. Between surveys, the global COVID-19 pandemic (2020–2022) disrupted school operations substantially, including closures, shifts to virtual instruction, and other interruptions to usual schooling.^11^ Given COVID-related resource reallocations and possible impacts on student performance and emotional well-being,^12^^,^^13^ reassessment of current health concern priorities and HPI availability in the post-pandemic context is critical to ensure implementation of relevant school-based health strategies.

Strengthening school health promotion requires understanding the current HPI landscape and its evolution in response to social, economic, and public health changes. Comparing Prome*SS* I and II helps determine whether efforts are progressing, stagnating, or becoming less equitable, which is critical information amid rising mental health concerns, increased screen use, and widening socioeconomic disparities. Without periodic assessments, emerging gaps or unintended policy consequences may go unnoticed.

Assessing implementation challenges is equally important, since HPI success depends on a school’s structural, financial, and human resources. Identifying these challenges is essential to ensuring HPIs are present and sustainable across diverse school contexts, particularly as schools rebuild capacity post-pandemic.

Herein, we report changes in perceived importance of health-related concerns and in HPI availability from 2016 to 2019, present new data on additional/emerging concerns and corresponding HPIs in 2023 to 2025 and examine perceived challenges to HPI implementation in the “pandemic” years (2020–2022).

## Methods

In 2016, we obtained permission from 32 of Quebec’s 69 school service centres (school boards), which supported 594 (33%) of the province’s 1795 public primary schools, to invite principals or proxies to participate in Prome*SS* I.^10^ In 2023, 35 centres, supporting 988 (52%) of 1881 schools, provided permission. A total of 171 schools (29% of eligible schools) participated in Prome*SS* I; 218 (22%) participated in Prome*SS* II. Only 25 took part in both waves. We used a repeat cross-sectional design to assess trends over time.

Prome*SS* received approval from the Centre hospitalier de l’Université de Montréal Ethics Review Board (2013-4130, CE 12.307; 2023-11168, 22.212-YP). Certificates were shared with service centres and principals. Service centres consented to the research team approaching schools, and each school informant provided consent.

### Questionnaire development

As described elsewhere,^10^ Prome*SS* I questionnaires were developed or adapted from previous studies and informed by implementation science frameworks,^9^^,^^14^^-^^19^ with some items created de novo. The instrument was vetted by an experienced school principal and pilot tested with 20 staff. In Prome*SS* II, new items were developed to capture emerging concerns, and the revised instrument was extensively pilot tested. To ensure comparability, items retained from Prome*SS* I were worded identically. New items were clearly identified and pretesting confirmed content validity. Differences in data collection mode (telephone in Prome*SS* I vs. online/paper in Prome*SS* II) may have influenced responses and are acknowledged as a potential source of variation. Further details and the full questionnaires are available online.^20^

### Data collection

Prome*SS* I data were collected from 2016/17 to 2018/19 in two-part telephone interviews (median length 52 minutes) in French or English. Principals/proxies provided data on their role, years of service, school characteristics, perceived priority of student health concerns, and HPI availability. Each school received $100. In Prome*SS* II, data were collected in 30 to 40-minute self-report questionnaires administered online using LimeSurvey on a secure server. In October 2023, participating schools received an invitation email with a personalized link, followed by reminders every two weeks for three months. In March 2024, remaining non-participants were contacted by telephone, and in June 2024 by a mailed questionnaire, with additional reminders in October and December 2024. Data collection ended in February 2025.

## Study variables

All public schools in Quebec with 30 or more students are ranked according to a school deprivation indicator,^21^^,^^22^ a composite of mothers without a high school diploma and parental employment status in the week preceding the census within the census area of each student’s residence.^23^ We classified schools as very advantaged (deciles 1–3), moderately advantaged (4–7) or disadvantaged (8–10). Catchment areas were classified by postal code^24^ as rural (< 1000 inhabitants), small (1000–29 999), medium (30 000–99 999) or large population centres (≥ 100 000).^25^ We also noted school service centres’ official language and total enrolment.

### Perceived importance of health concerns

Perceived importance of health concerns was assessed by: “In the past year, how important was each of the following health-related issues for students?” Importance was defined as warranting special attention. Prome*SS* I informants responded to 13 health concerns identified by the Institut national de santé publique du Québec.^26^ Concerns about unhealthy eating, inadequate sleep, physical inactivity, dental problems, infectious diseases/parasites, personal hygiene, ADHD, aggressive/disruptive behaviour, and lack of respect for safety were queried for all grades; bullying/cyberbullying/exclusion and cigarette smoking were queried for grades 3 to 6; mental health and puberty for grades 5 to 6.

Prome*SS* II informants responded to these 13 plus 15 additional concerns: screen time, social media use, unsafe technology use, sedentary behaviour, respiratory viruses, e-cigarettes/vaping, alcohol, cannabis, other drugs, learning difficulties, body image, self-harm/suicidal thoughts, eating disorders, trauma exposure, and food insecurity/poverty. Concerns were identified through expert consultation, provincial reports, and pilot testing. Response choices were recoded as important (important, very important, extremely important) vs. not important (not very important, not important).

### HPI availability

HPIs were defined as curriculum-complementary activities offered to all students during class at no cost, with mandatory attendance. HPI availability was measured by: “In the past year, has your school offered any HPIs…?, followed in Prome*SS* I by eight topics. The Ministry of Education directs schools to implement HPIs on physical activity/active living,^27^ oral health,^28^ sex education,^29^ and bullying/cyberbullying/exclusion.^30^ HPIs on mental health and well-being, healthy eating, cigarette smoking, and personal safety/injury prevention are offered at the school’s discretion. Prome*SS* II included 12 additional discretionary topics: screen time, unsafe technology use, sedentary behaviour, respiratory viruses, vaping, alcohol, cannabis, other drug use, sleep, trauma, behaviour regulation, and environmental awareness (reflecting increasing recognition of its importance).^31^

### Challenges to HPI implementation

Challenges to HPI implementation were assessed with “In the past 3 years, how important were each of the following challenges….?” Response options were not at all, a bit, or very challenging. Issues included several derived from implementation science frameworks:^17^^-^^19^ lack of staff time, inadequate support, school staff unable to solve implementation issues, inadequate funding, space constraints, poor adaptation of the HPI to student needs, and lack of commitment/interest among parents, teachers, or students, plus COVID-19 restrictions, lack of guidance, and low priority for student health.

## Data analysis

All analyses were descriptive; we report percentages. We calculated differences between Prome*SS* I and II in percentage of informants who identified concerns as important and whose schools implemented corresponding HPIs. Following STROBE guidelines for observational studies, we did not conduct significance testing because random sampling was not used, the sample was underpowered for school-level comparisons, and small cell sizes made inferential testing inappropriate.^32^

## Results

Table 1 compares Prome*SS* I and II schools with all Quebec public primary schools from 2017 to 2025. The Prome*SS* samples are broadly representative, with modest overrepresentation of rural and smaller schools, in Prome*SS* II.

**Table 1:** Comparison of schools participating in Prome*SS* I and Prome*SS* II with primary schools in Quebec, Canada

**School characteristic**	**Prome*SS* I** **2016–2019** **(n = 171)**	**Prome*SS* II** **2023–2025** **(n = 218)**	**Quebec** **2017–2018** **(n = 1795)^b^**	**Quebec** **2023–2024** **(n = 1881)^b^**
School deprivation^a^				
Disadvantaged (%)	36	41	38	35
Moderately advantaged (%)	44	40	39	41
Highly advantaged (%)	21	19	24	24
Rural service area^c^ (%)	40	37	30	29
French language school (%)	83	89	90	90
Median number of students per school	267	249	259	280

^a^ Based on the Indice de milieu socioéconomique (Socioeconomic background index [IMSE]),^21^^,^^22^ a composite score reflecting the proportion of mothers without a high school diploma and the proportion of unemployed parents in the week preceding the census within the census area of the student’s residence.^23^

^b^ The “n” for comparator schools is the number to which an IMSE was assigned.

^c^ Rural service areas have fewer than 1000 inhabitants.^25^

## Changes between Prome*SS* I and II

Table 2 summarizes changes in perceived importance of student health concerns and HPI availability.

**Table 2:** Changes between Prome*SS* I (2017–2019) and Prome*SS* II (2023–2025) primary schools in perceived importance of health concerns and HPI availability

**Health-related concern**	**Prome*SS* I** **(n = 171)** **%**	**Prome*SS* II** **Perceived importance^a^** **(n = 194)** **HPI availability** **(n = 165)** **%**	**Change**
Unhealthy eating			
Perceived importance	51	67	16% increase
HPI availability	70	65	5% decrease
Inadequate sleep			
Perceived importance	63	78	15% increase
HPI availability	n/a^c^	59	n/a
Lack of physical activity^b^			
Perceived importance	51	64	13% increase
HPI availability	89	76	13% decrease
Dental problems^b^			
Perceived importance	30	24	6% decrease
HPI availability	94	75	19% decrease
Infectious diseases/parasites			
Perceived importance	46	36	10% decrease
HPI availability	n/a	n/a	n/a
Personal hygiene			
Perceived importance	36	36	0% change
HPI availability	n/a	n/a	n/a
Problems with mental health			
Perceived importance	65	77	12% increase
HPI availability	42	54	12% increase
Attention deficit hyperactivity disorder			
Perceived importance	79	91	12% increase
HPI availability	n/a	n/a	n/a
Aggressive/disruptive behaviour			
Perceived importance	68	72	4% increase
HPI availability	n/a	84	n/a
Bullying/cyberbullying/exclusion^b^			
Perceived importance	60	54	6% decrease
HPI availability	90	91	1% increase
Lack of respect for personal safety			
Perceived importance	61	65	4% increase
HPI availability	45	58	13% increase
Concerns about puberty^b^			
Perceived importance	54	37	17% decrease
HPI availability	85	90	5% increase
Cigarette smoking			
Perceived importance	5	6	1% increase
HPI availability	9	13	4% increase

**Abbreviation:** HPI, health-promoting intervention.

^a^ The Prome*SS* II questionnaire contained 72 stems, most with numerous individual items. Stem #32 addressed the importance of health issues (29 items) and stem #33 addressed HPI availability (21 items). The higher the item count, the lower the number of informants who responded.

^b^ Ministry of Education mandates that schools offer HPIs related to concern.

^c^ n/a = data not available because availability of related HPIs was not queried.

Perceived importance increased for unhealthy eating, inadequate sleep, and physical inactivity. Concerns about infectious diseases/parasites and dental problems declined. Despite ministry mandates, HPI availability decreased for physical activity, dental health, and unhealthy eating. Sleep-related HPI availability was queried only in Prome*SS* II.

Perceived importance and HPI availability increased for mental health problems. ADHD showed a large increase in perceived importance; related HPI availability was not assessed. Perceived importance of aggressive/disruptive behaviour and lack of respect for personal safety rose slightly, with substantial increases in relevant HPIs, including newly reported interventions for aggressive behaviour. Perceived importance of bullying/cyberbullying and exclusion declined slightly, while related HPI availability remained stable. Concern about puberty decreased markedly, while HPI availability increased slightly, consistent with mandated sexual health education. Cigarette smoking remained a low-priority concern, with few related HPIs implemented.

## Prome*SS* II: Priority health concerns and HPI availability

Table 3 shows Prome*SS* II informants identifying concerns as important and reporting HPI availability. Screen time and social media use were the most frequently cited concerns. HPIs addressing screentime were available in only three schools while HPIs addressing unsafe technology use were available in three-quarters of schools. About half cited sedentary behaviour and one-third cited respiratory viruses, with HPI availability lagging perceived importance by 9% and 13%, respectively. Fewer than 10% identified e-cigarettes, alcohol, cannabis, or drugs as concerns, and 10% to 15% of schools offered related HPIs.

**Table 3:** Proportion of primary schools that rated specific health-related concerns as important among their student population and reported implementing health-promoting interventions addressing specific concerns in the past year (Prome*SS* II)

**Health-related concerns**	**Perceived importance** **(n = 194)** **%**	**HPI availability** **(n = 165)** **%**
Screen time	98	1.8
Use of social media	89	n/a
Unsafe use of technology	52	75
Sedentary behaviour	51	42
Respiratory viruses^a^	37	24
E-cigarettes (vaping)	7	15
Alcohol use	5	10
Cannabis use	5	10
Drug use (excluding cannabis use)	4	12
Learning difficulties	92	n/a
Concerns about body image	45	n/a
Self-harm, suicidal thoughts	24	n/a
Eating disorders	16	n/a
Exposure to trauma^b^	39	31
Food insecurity/poverty	47	n/a
Environmental awareness^c^	n/a	74

**Abbreviation:** HPI, health-promoting intervention.

**Note:** n/a = data were not collected.

^a^ Respiratory virus (e.g. RSV, flu, common cold, COVID-19).

^b^ Exposure to trauma examples: traumatic situations as a witness or victim (e.g. mistreatment, abuse, neglect, violence).

^c^ Environmental awareness (e.g. Earth Day, air quality, quality of drinking water).

Learning difficulties were widely reported. Body image concerns and trauma exposure were cited by over one-third; self-harm/suicidal thoughts by one-quarter; and eating disorders by 16%. Availability data were not collected for most concerns, although trauma-related HPIs were reported by about one-third. Almost half cited food insecurity or poverty as important, and environmental awareness was addressed by HPIs in three-quarters of schools.

## Perceived challenges to HPI implementation

Among 165 Prome*SS* II schools, most identified lack of staff time and almost half identified inadequate funding as very important barriers to implementation (Table 4). Fewer cited COVID-19 restrictions, parental disengagement, or insufficient support as major obstacles, although over one-third still found them challenging. Most did not consider space constraints or lack of information important. Over 85% reported few or no challenges related to teacher or student interest, engagement, or motivation. Nearly all reported minimal difficulty with institutional priority for student health or HPI adaptability. Overall, time and funding, not interest, are the main bottlenecks.

**Table 4:** Perceived challenges to implementing school-based health-promoting interventions in the past three years (n = 165)

**Issue^a^**	**Challenging**		
	**Not at all** **%**	**A bit** **%**	**Very** **%**
Lack of staff time to implement HPI	5	33	62
Inadequate funding	12	43	45
Restrictions due to COVID-19	40	21	39
Lack of commitment or interest among parents	21	44	36
Inadequate support to implement HPI	13	52	35
Lack of space	38	37	25
Lack of information on types of activities to implement	16	59	24
Lack of commitment or interest among teachers	22	65	13
School staff unable to solve implementation issues	38	50	12
Lack of commitment or interest among students	35	53	12
Low priority of student health	57	37	6
HPI was not well adapted to student needs	55	41	4

**Abbreviation:** HPI, health-promoting intervention.

^a^ Listed in order of importance (i.e. % “very challenging”).

## Discussion

Our findings reveal gaps from 2016 to 2025 between student health concerns and HPI availability. Mental health was the most frequently cited concern; while mental health HPIs increased, a large deficit between perceived importance and availability persisted. Comparable gaps were observed for physical inactivity, unhealthy eating, and inadequate sleep. Excessive screen time also emerged as a prominent concern. These results underscore the increasing complexity of implementing school-based HPIs amid evolving student needs and constrained resources. Even as schools expand HPIs, the pace of growth has not kept up with the rapid rise in health concerns. Consistent with national and international research on health-promoting schools, which has documented similar implementation gaps and resource pressures,^1^ our results reinforce these challenges while extending the evidence base using province-wide trend data. This contribution is unique in the Canadian context, where few systems track alignment at scale. Addressing these gaps will require additional resources and more flexible, targeted approaches that reflect shifting priorities.

### Shifting priorities and uneven implementation

Consistent with international and Canadian studies, we observed increased concern about student mental health.^33^^,^^34^ The 12% increase in mental health HPI availability aligns with national trends toward expanding school-based initiatives.^35^ However, a persistent implementation gap (over a quarter of concerned schools reported no related HPI) may reflect systemic barriers, including insufficient staff time, funding instability, limited training opportunities, fragmented school–public health coordination, and weak monitoring systems.^36^ Overall, this gap underscores the need for targeted support strategies, such as dedicated funding streams and HPI coordinator roles.

HPIs addressing physical activity declined by 13% despite rising concerns and longstanding mandates. This reflects national and global trends, where mandated programs face implementation fatigue due to staffing shortages and competing demands.^37^^,^^38^ Nutrition-related HPIs remained static, and dental health HPIs declined, despite being mandated.^28^ These trends suggest that mandates alone do not ensure sustained delivery, when perceived relevance is low or resources are limited. By contrast, sex education remained widely available (> 90%) despite reduced concerns, suggesting some HPIs are maintained for compliance rather than responsiveness.

The mismatch between mandated HPIs and school-identified priorities signals a need to reassess policies. Schools appear most responsive to urgent, modifiable concerns, as seen in the widespread uptake of behaviour-regulation programs despite no mandate. Conversely, perceived low-relevance HPIs may persist. The interconnected nature and common causes of issues underscores the limitations of siloed programs. An integrated, multitopic model may be more effective under resource strain. Embedding multiple health-promotion components into routine operations increases sustainability,^39^ and integrated interventions addressing dietary habits, green‐space use and mental health showed improved outcomes.^40^

### Gaps in program coverage for emerging issues

The discrepancy between schools identifying screen time (98%) and sleep (78%) as concerns and those implementing related HPIs (1.8% and 59%) is striking. These intertwined behaviours are key to development and chronic disease prevention,^41^^-^^43^ yet under-addressed. Our findings highlight a need for integrated approaches addressing digital health, sleep hygiene, and mental well-being, rather than isolated programs, echoing calls for coordinated interventions.^43^

Schools also reported increased concern about body image, disordered eating, and self-harm, issues requiring not only HPIs but also access to mental health professionals. In contrast, cannabis use and vaping were rarely cited, reflecting age patterns of substance use initiation.^44^

Three high-priority concerns fall largely outside HPI scope. ADHD requires individualized, multisystem supports,^45^^-^^47^ some partially met through PSEs, but full intervention demands cross-sector collaboration. Learning difficulties likely reflect COVID-19 pandemic–related learning losses,^48^ and food insecurity may be best addressed by expanding universal school meal programs.^49^ As of October 2025, provincial, territorial and Indigenous governments established bilateral agreements with and receive funding from Canada’s National School Food Program to expand and strengthen school meal services nationwide.^50^

Despite these gaps, few schools reported low engagement from staff, students, or families, suggesting strong support for HPIs despite limited resources. The lack of programs targeting sleep, screen time, and food insecurity may reflect limited models or capacity.

### Policy and practice implications

Staff shortages, lack of time, and limited funding were the most important challenges to HPI implementation and were worsened by the pandemic, which disrupted programs and intensified student needs. Consistent with prior research, our findings confirm well-established barriers. However, the novelty of Prome*SS* lies in providing population-level trend data on alignment between perceived health priorities and HPI availability across a wide range of topics.

Our findings suggest that school leaders are motivated and aware, but existing structures are insufficient. Stakeholders may consider: (i) re-visiting mandates; (ii) investing in HPIs that address multiple health issues; (iii) partnering with public health and community organizations; and (iv) allocating stable funding and dedicated staff, including school health promotion facilitators, particularly in underserved schools; and (v) establishing monitoring systems tracking alignment between concerns and HPI coverage.

## Strengths and limitations

This study draws on a large, diverse sample that is broadly representative of Quebec primary schools in IMSE categories and school size and includes data from two time points spanning pre- and post-pandemic. It is among the first to document both health priorities and HPI availability trends using a validated tool. Self-reports from a single informant may have resulted in misclassification. Social desirability bias may be introduced because principals are reporting on staff under their supervision. In addition, because data were reported exclusively by school staff, the study does not incorporate the first-person perspectives of students themselves, despite the focus on student health priorities. Only 25 schools participated in both Prome*SS* waves, limiting direct comparison. The modest sample size and descriptive statistics may limit generalizability. Nevertheless, the representativeness of our samples in both waves supports cautious interpretation of our findings as system-level patterns. This analysis does not explicitly apply an equity lens. Although IMSE captures school-level deprivation, finer-grained analyses (e.g. race/ethnicity, disability, intersectionality) were not possible. Finally, repeat cross-sectional studies cannot assess causality.

## Future research

More research is needed to examine factors influencing HPI uptake when mandates exist but delivery lags. Longitudinal analyses of schools participating in both Prome*SS* waves could clarify sustainability patterns. Qualitative work may reveal how schools weigh urgency and resource trade-offs. Future studies should assess equity and include student perspectives. The impact of new policies, such as the 2025 cell phone ban, should be evaluated.

## Conclusion

From 2016 to 2025, Quebec primary schools faced rising student health concerns alongside widening gaps in capacity to respond. While mental health promotion advanced, other domains remain neglected. Our findings call for updated policies, integrated strategies, and sustained investment. Strengthening these supports is essential to ensure responsive, equitable, and effective health promotion during this post-pandemic period of recovery and reform. Addressing these gaps will likely require sustained intersectoral collaboration.

## Data Availability

None.
